# A Rare Case of Appendiceal Metastasis From Prostate Adenocarcinoma in an Asymptomatic Patient With Biochemical Recurrence

**DOI:** 10.1002/ccr3.73839

**Published:** 2026-11-03

**Authors:** Antonio Muina, Mohamed Javid Raja Iyub, Michael Toussaint, Murugesan Manoharan

**Affiliations:** ^1^ St. George University School of Medicine St. George's Grenada; ^2^ Miami Cancer Institute Baptist Health South Florida Miami Florida USA; ^3^ Herbert Wertheim College of Medicine Florida International University Miami Florida USA

**Keywords:** adenocarcinoma, appendiceal metastasis, biochemical recurrence, metastatic prostate cancer, prostate cancer

## Abstract

We report a rare case of appendiceal metastasis from prostate adenocarcinoma in a 73‐year‐old male with a prior Gleason score 7 prostate cancer, initially treated with radical prostatectomy and adjuvant radiation in 2014. The patient presented in 2025 with a rising prostate‐specific antigen (PSA) and modest prostate‐specific membrane antigen (PSMA) uptake on positron emission tomography/computed tomography (PET/CT) at the tip of the appendix. Magnetic resonance imaging (MRI) demonstrated a corresponding appendiceal lesion with restricted diffusion and enhancement. Pathology following laparoscopic appendectomy confirmed metastatic prostate adenocarcinoma involving the appendix and periappendiceal lymph nodes. This case highlights the importance of correlating atypical PSMA PET/CT findings with anatomic imaging and, when needed, histologic confirmation in patients with biochemical recurrence of prostate cancer.

## Key Clinical Message

Metastasis of prostate adenocarcinoma to the appendix is a rare presentation and may be identified in an asymptomatic patient during evaluation of biochemical recurrence. Because modest PSMA uptake at the appendix can be nonspecific, correlation with anatomic imaging and histologic confirmation may be important when the finding would alter treatment planning.

## 1 Introduction

Prostate cancer is the fourth most common cancer worldwide and the eighth most common cause of cancer‐related mortality [1]. The 5‐year survival rate for prostate cancer with distant metastasis is only 30% [2, 3, 4]. Prostate cancer commonly metastasizes to bone, lymph nodes, and occasionally the liver or lungs. Metastasis of prostate adenocarcinoma to the appendix is exceedingly rare, with a minimal number of documented cases available worldwide [5, 6, 7]. This report presents a unique case of histopathologically confirmed appendiceal involvement, underscoring the need for high clinical suspicion in the face of atypical imaging findings.

## 2 Case History/Examination

A 73‐year‐old male with a history of prostate adenocarcinoma, initially diagnosed in 2013 with a Gleason score of 7 (3 + 4), presented for evaluation due to rising prostate‐specific antigen (PSA) levels during follow‐up. He was previously treated with radical prostatectomy and adjuvant radiation therapy in 2014. At initial diagnosis, his prostate cancer was staged as T3aN0M0. The Eastern Cooperative Oncology Group (ECOG) performance status was 0, and the PSA at the time of re‐evaluation was 7.2 ng/mL. The patient did not have any classic features of appendicitis and reported no urinary symptoms. He also denied back pain, bone pain, weight loss, fever, fatigue, or other constitutional symptoms. Medical history included Type 2 diabetes mellitus, fatty liver disease, hypertension, Barrett's esophagus, diverticulosis, former smoking, rising PSA levels, prior skin cancer, and previous androgen deprivation therapy (ADT).

## 3 Differential Diagnosis, Investigations and Treatment

Prostate‐specific membrane antigen positron emission tomography/computed tomography (PSMA PET/CT) performed in March 2025 showed a 1.4‐cm soft tissue density at the tip of the appendix with modest PSMA uptake (maximum standardized uptake value [SUVmax] of 3.1) and a small focus of mildly increased PSMA uptake in the posterior left 7th rib (Figures 1 and 2). Magnetic resonance imaging (MRI) of the abdomen confirmed a T2‐intermediate signal lesion at the appendiceal tip with restricted diffusion and enhancement. A bone scan was performed and revealed focal uptake in the 7th rib and iliac bone, which favored benign osseous abnormalities. Although low‐level PSMA uptake can be nonspecific, and physiologic appendiceal uptake has been reported [8], the discrete 1.4‐cm lesion and corresponding MRI findings raised concern in the setting of biochemical recurrence. Following discussion of the suspicious lesion with the patient, a referral was made to a colorectal surgeon. The decision to proceed with surgery was made after discussion with the patient and colorectal surgery; the case was not formally reviewed at a multidisciplinary tumor board. Because the diagnosis would influence systemic treatment planning and the appendiceal lesion remained indeterminate, tissue diagnosis was pursued. Systemic therapy consisting of ADT in combination with a second‐generation antiandrogen was also discussed, should the lesion be confirmed to be metastatic prostate cancer. Laparoscopic appendectomy with partial cecectomy was performed in May 2025.

**FIGURE 1:**
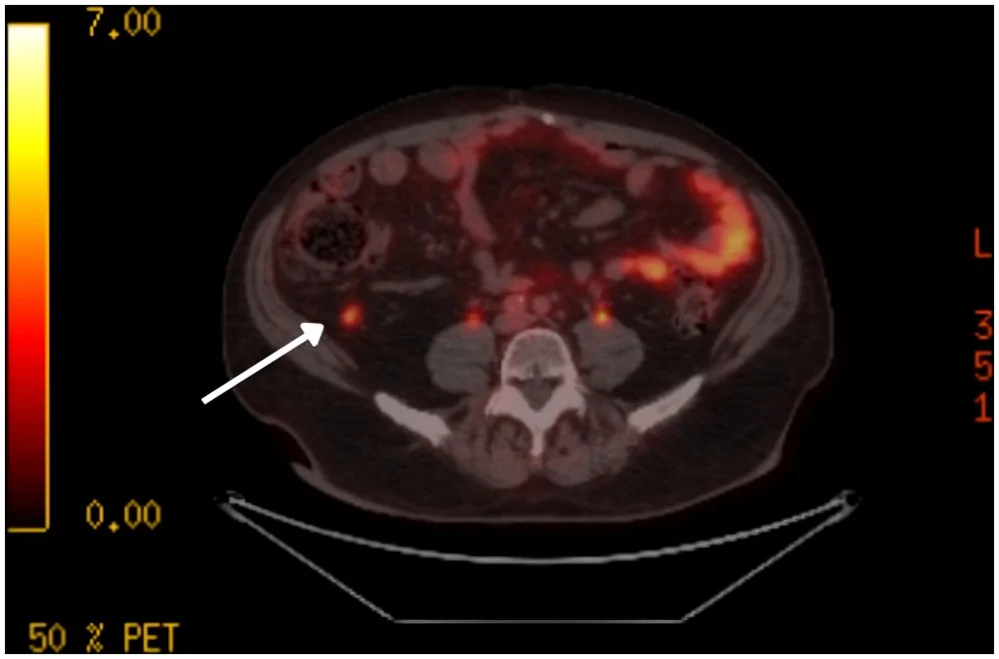
PET/CT (cross section) demonstrating increased PSMA uptake in the appendix (arrow).

**FIGURE 2:**
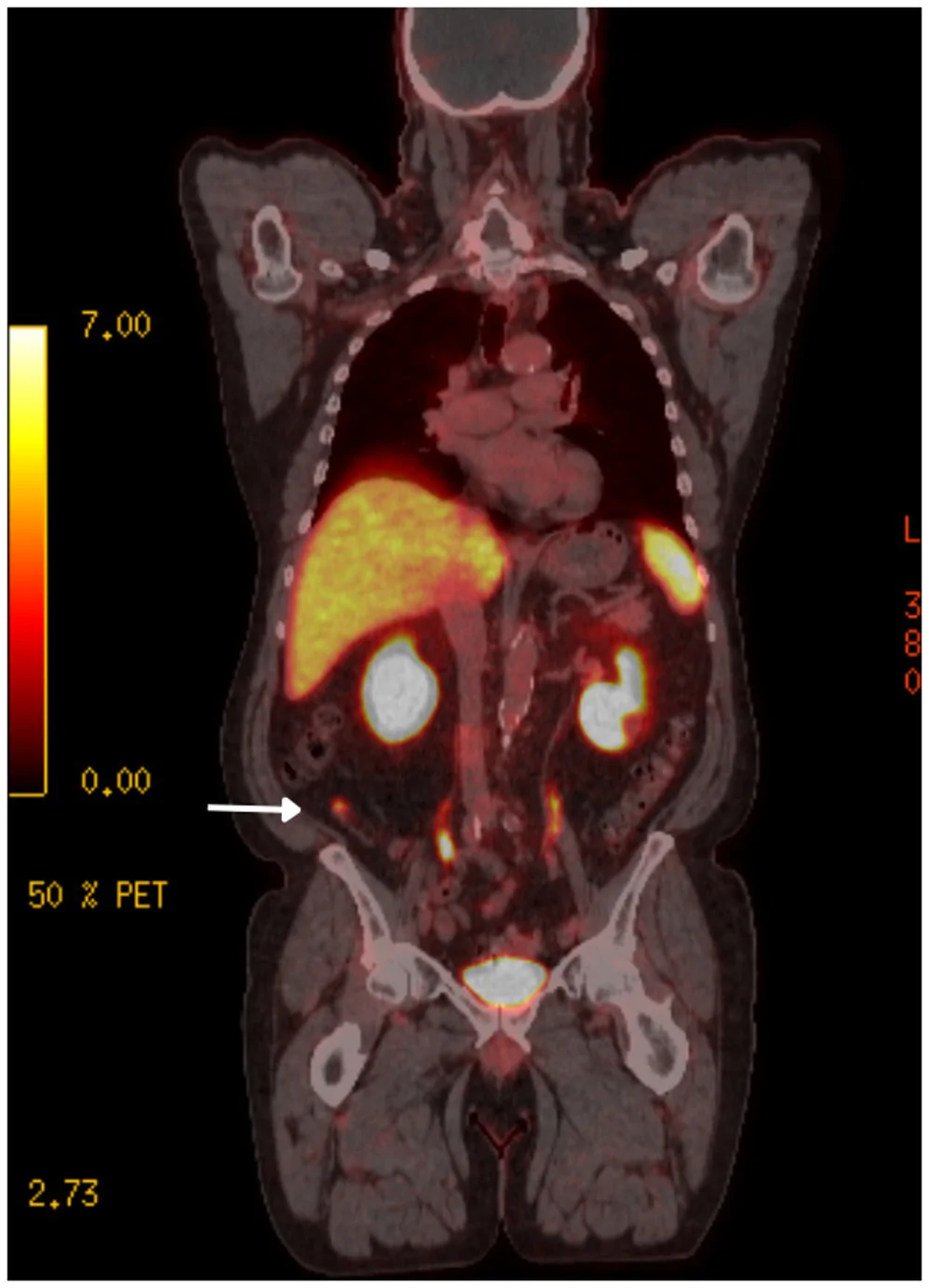
PET/CT (coronal section) demonstrating increased PSMA uptake in the appendix (arrow).

## 4 Conclusion and Results (Outcome and Follow‐Up)

Pathology findings confirmed metastatic prostate adenocarcinoma. The lesion involved the full‐thickness appendiceal wall and serosa, and two of four periappendiceal lymph nodes were positive. The appendix and periappendiceal lymph nodes represented the only histologically confirmed sites of metastatic disease. A positive stapled resection margin was also identified. The predominant tumor focus was at the appendiceal tip, with patchy involvement elsewhere. No additional surgical resection was pursued, and serial follow‐up imaging has shown no local recurrence at the operative site. Immunohistochemistry showed NKX3.1 (positive), CK7/CK20/CDX2 (negative), synaptophysin (patchy positive), and prostatic acid phosphatase (PSAP; essentially negative). The retained NKX3.1 expression supported prostatic origin, while patchy synaptophysin staining, with essentially negative PSAP, was suggestive of focal neuroendocrine differentiation [9, 10]. PSA, measured 5 weeks after surgery in June 2025, was 6.3 ng/mL. A follow‐up PET/CT carried out in July 2025 showed the same PSMA uptake in the posterior left 7th rib as well as a nonspecific focus of increased PSMA uptake to the left of the midline along the distal left inguinal region, for which a metastatic implant could not initially be excluded. Magnetic resonance imaging assessment and close follow‐up were suggested. Review of prior imaging showed that the left inguinal finding had been present since 2017. Both the left 7th rib and left inguinal findings remained stable during follow‐up and were interpreted by radiology as sclerotic changes without suspicion for metastatic disease. At the end of July 2025, the PSA remained elevated at 6.4 ng/mL. The persistent PSA elevation suggested ongoing prostate cancer activity despite removal of the appendiceal lesion. ADT was subsequently restarted, and the most recent PSA decreased to 0.4 ng/mL.

## 5 Discussion

Our case report illustrates a highly uncommon site of metastasis for prostate cancer, with appendiceal involvement confirmed through imaging and pathology. An important distinction in this case is that the patient was entirely asymptomatic, and the lesion was identified during evaluation of biochemical recurrence with PSMA PET/CT rather than after presenting with acute appendicitis. Metastasis of prostate adenocarcinoma to the appendix is very uncommon [6, 7]. Most appendiceal metastases present as acute appendicitis, either clinically or radiographically [11]. In one case reported from Australia, an 82‐year‐old male with known bone metastases from prostate cancer presented with appendicitis. Subsequent appendectomy and histopathologic examination confirmed metastatic prostatic adenocarcinoma in the appendix [11]. Similarly, a 72‐year‐old man presented with classical acute appendicitis symptoms and underwent appendectomy, with immunohistochemical staining revealing strong PSA positivity in the appendiceal metastasis [12]. In contrast, our patient had no appendicitis symptoms, and the lesion was detected incidentally during evaluation of biochemical recurrence. These reports highlight the diagnostic challenge since standard CT findings often cannot distinguish benign causes from metastatic lesions.

A case report reviewing acute appendicitis secondary to metastatic prostatic adenocarcinoma in a 66‐year‐old male noted that metastases from various neoplasms accounted for around 0.1% of all appendectomy specimens [13]. A systematic review of 78 cases of appendicitis secondary to metastatic disease showed breast, lung, gastric, and prostate cancers as the most common primary sources. Among prostate‐origin cases (8 patients), approximately 37% (3 patients) presented with complicated appendicitis. Among all the metastatic cases that presented with appendicitis, 46% of patients presented with complicated appendicitis, compared with 19% presenting with perforation in typical nonmetastatic cases [6, 14]. In our patient, the appendiceal PSMA uptake was modest and therefore not diagnostic in isolation. Physiologic appendiceal PSMA uptake has been reported and may mimic disease [8]. However, the combination of a discrete 1.4‐cm lesion, MRI enhancement, and restricted diffusion increased suspicion and supported surgical evaluation. The left 7th rib and left inguinal findings remained stable on serial imaging and were interpreted by radiology as benign sclerotic changes rather than as metastatic disease. The appendiceal lesion and periappendiceal lymph nodes therefore remained the only histologically confirmed metastatic sites. Immunohistochemical analysis is important for confirming metastatic prostate origin, especially in distinguishing these lesions from primary appendiceal neoplasms [13, 15]. NKX3.1 is particularly specific for prostate tissue and was positive in our patient's specimen, reinforcing the diagnosis. The patchy synaptophysin positivity with essentially negative PSAP suggests focal neuroendocrine differentiation rather than, by itself, a separate neuroendocrine carcinoma [9, 10].

Our case report highlights several important clinical implications. One is diagnostic vigilance; in prostate cancer patients with rising PSA and atypical abdominal imaging, appendiceal lesions warrant careful evaluation, whereas modest PSMA uptake should be interpreted together with anatomic imaging. Another is surgical strategy, as tissue diagnosis may be useful when an indeterminate lesion would influence systemic treatment planning. In this patient, appendectomy provided definitive diagnosis and local control. Although the stapled resection margin was positive, the predominant tumor focus was at the appendiceal tip with patchy involvement elsewhere, and no additional surgical resection was pursued. Serial imaging has shown no local recurrence at the operative site. The PSA remained in the 6.3–6.4 ng/mL range after surgery, indicating persistent prostate cancer activity despite removal of the appendiceal lesion and raising the possibility of residual microscopic or otherwise occult disease. No additional metastatic site has been confirmed. Because of the persistent biochemical recurrence, ADT was restarted, and the most recent PSA decreased to 0.4 ng/mL. As the literature is limited to case reports and small series, with no standardized management algorithm, there is a need for multicenter patient registries to better characterize incidence, optimal management strategies, and long‐term outcomes in metastatic appendiceal lesions. The limited duration of follow‐up in this case should also be acknowledged.

Metastatic spread of prostate cancer to the appendix is exceedingly rare and may occur without symptoms of appendicitis. In patients with biochemical recurrence and atypical PSMA PET/CT findings, modest uptake should be correlated with structural and other imaging findings rather than interpreted in isolation. When the diagnosis would influence treatment, histologic confirmation may help guide subsequent management. This case supports the role of comprehensive multimodal assessment and continued biochemical and imaging surveillance following treatment.

## Author Contributions

**Antonio Muina:** conceptualization, data curation, resources, visualization, writing – original draft, writing – review and editing. **Mohamed Javid Raja Iyub:** methodology, project administration, supervision, visualization, writing – original draft, writing – review and editing. **Michael Toussaint:** methodology, writing – original draft, writing – review and editing. **Murugesan Manoharan:** formal analysis, investigation, project administration, resources, software, supervision, validation, writing – original draft, writing – review and editing.

## Funding

The authors have nothing to report.

## Consent

Written informed consent was obtained from the patient for publication of this case report and accompanying images in accordance with the journal's patient consent policy.

## Conflicts of Interest

The authors declare no conflicts of interest.

## Data Availability

The data that support the findings of this study are available on request from the corresponding author. The data are not publicly available due to privacy or ethical restrictions.
